# Kondo-like transport and magnetic field effect of charge carrier fluctuations in granular aluminum oxide thin films

**DOI:** 10.1038/s41598-018-32298-1

**Published:** 2018-09-17

**Authors:** C. Barone, H. Rotzinger, C. Mauro, D. Dorer, J. Münzberg, A. V. Ustinov, S. Pagano

**Affiliations:** 1 Dipartimento di Fisica “E.R. Caianiello” and CNR-SPIN Salerno, Università di Salerno, I-84084 Fisciano, Salerno, Italy; 2 Physikalisches Institut, Karlsruhe Institute of Technology, 76131 Karlsruhe, Germany; 3 Dipartimento di Ingegneria, Università del Sannio, I-82100 Benevento, Italy; 4 Russian Quantum Center, National University of Science and Technology MISIS, 119049 Moscow, Russia

## Abstract

Granular aluminum oxide is an attractive material for superconducting quantum electronics. However, its low-temperature normal state transport properties are still not fully understood, while they could be related to the unconventional phenomenon of the superconductivity in this material. In order to obtain useful information on this aspect, a detailed study of charge carrier fluctuations has been performed in granular aluminum oxide films. The results of electric noise measurements indicate the presence of a Kondo-type spin-flip scattering mechanism for the conducting electrons in the normal state, at low temperatures. Moreover, the magnetic field dependence of the noise amplitude suggests that interface magnetic moments are the main source of fluctuations. The identification of the nature of fluctuation processes is a mandatory requirement for the improvement of quality and performance of quantum devices.

## Introduction

Quantum computation is one of the most attracting research fields, due to the possibility of implementing computational machines much more performant than classical computers^1^. In the last yeas, great progresses have been achieved both from a theoretical and technological side^2^, with the realization of proof-of-concept algorithms on quantum processors^3,4^.

Among the most useful materials for quantum electronics, superconductors allow in principle the realization of scalable quantum integrated circuits. In particular, aluminum has already shown its potentials thanks to its low surface dielectric loss tangent^5^ and to the growth of a controllable oxide barrier between the electrodes of Josephson junctions. For quantum circuits where an enhanced kinetic inductance is of interest, granular aluminum oxide (AlO_*x*_) is, among other materials, an interesting candidate. It offers a good combination of a low microwave loss superconducting state, as well as a very high normal state sheet resistance^6^. However, several issues regarding the normal state transport properties of AlO_*x*_ thin films deserve to be still understood, while they can have a relation with the unconventional superconducting mechanism in this type of material.

More in details, the origin of a metal-to-insulator transition, frequently observed above the superconducting critical temperature in AlO_*x*_ samples, is still a matter of debate and different theoretical interpretations have been proposed. As a first hypothesis, weak-localization effects, arising from electron interference, were considered responsible for the reduction of the low-temperature normal state conductivity of percolative superconducting aluminum films^7^. Later on, the low-temperature normal state resistance behaviour of granular aluminum films was explained in terms of a Kondo-like transport mechanism^8^. Recently, a low-temperature Mott transition has been identified in granular aluminum, as a consequence of a nanosize grains decoupling induced by a progressive reduction of the intergrain tunneling probability^9^. All these models have a solid physical background and seem to satisfactorily apply to the case of granular metals. Therefore, on the basis of standard electric transport investigations, it is very difficult to disentangle between them. A more sensitive experimental technique is then required.

In this respect, electric noise spectroscopy has proved to be a very effective and efficient method for studying the kinetic processes of the charge carriers in several systems. As examples, it has strongly contributed to clarify unsolved fundamental questions regarding the electronic properties of manganites^10,11^, iron-based superconductors^12,13^, carbon nanotube composites^14^, and perovskite-based solar cells^15^. Moreover, the study and control of fluctuation mechanisms plays a crucial role to improve the efficiency of the future devices for quantum information processing^16^. Indeed, it is evident that an improvement of the quality of quantum computing devices is obtained by isolating the quantum bits (qubits) from external decoherence sources. The identification of the origin of charge carriers fluctuations is, therefore, essential for the reduction of these sources of decoherence^16^.

In view of all these considerations, detailed DC electric and magneto-transport measurements, as well as voltage-noise analysis, of granular aluminum thin films are here reported. Different theoretical models have been considered and analyzed, in order to reproduce the experimental findings. The results obtained contribute to shed light on the normal state conductivity mechanisms, providing, also, interesting information that could help for a better understanding of the unconventional phenomenon of the superconductivity in granular films.

## Results and Discussion

### DC electrical transport measurements

**Figure 1:**
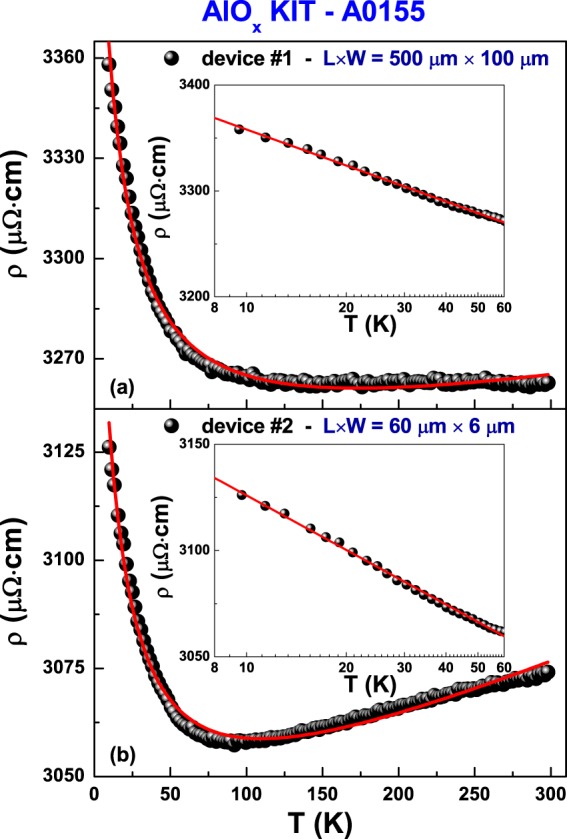
Resistivity versus temperature plots. The data refer to the large-strip device #1 (**a**) and to the small-strip device #2 (**b**). The red solid lines are the best fitting curves using equation (1) with the Kondo resistivity as the “insulating” term *ρ*_*I*_. The enlargements of the low-temperature region (8–60 K) are shown as insets with a logarithmic temperature scale.

The resistivity temperature dependence is shown in Fig. 1 for the large-strip device (device #1 in Fig. 1(a)) and for the small-strip device (device #2 in Fig. 1(b)). An evident increase at low temperatures is observed, in agreement with earlier findings in similar resistive films^7,8^. Moreover, a characteristic minimum is clearly found only for the smaller device, see Fig. 1(b). In general, the *ρ* vs *T* behaviour can be modeled by two additive contributions: a metal-like and a generic “insulating” term as

1 $$\rho (T)=A{T}^{n}+{\rho }_{I}(T),$$

where the metallic resistivity *AT*^*n*^ is a power law with *n* = 1, 2, or 5. In particular: *n* = 1 indicates the presence of charged-impurity resistivity contributions^17,18^, *n* = 2 is characteristic of the standard metallic Fermi-liquid behaviour, *n* = 5 reveals resistivity contributions due to lattice vibration^19^. The “insulating” term *ρ*_*I*_ can be analyzed in terms of different models, as^20^: (I) Mott variable-range hopping (VRH), (II) Efros-Shklovskii (ES) localization, (III) two-dimensional (2D) weak localization (WL), (IV) three-dimensional (3D) weak localization (WL), (V) fluctuation-induced tunneling (FIT), and (VI) Kondo effect. The explicit temperature dependencies are reported in Tables 1 and 2. All these models are generally able to reproduce well the data, giving very similar values of statistical parameters (i.e., reduced *χ*^2^ and coefficient of determination *r*^2^) as shown in Tables 1 and 2, where the coefficients related to the metallic resistivity term are also reported. The various parameters describing the *ρ*_*I*_ term are discussed in details in ref.^20^. As an example, the curve corresponding to the Kondo-resistivity term is shown in Fig. 1 as a red solid line. The good agreement between theory and data is also shown by the enlargement of the low-temperature region, reported in the insets of Fig. 1 with a logarithmic temperature scale.

**Table 1:** Comparison between different theoretical models of *ρ* vs *T* curves for device #1.

Model	*A* (*μ*Ω*cmK*^−*n*^)	*n*	*ρ*_*I*_(*T*)-dependence	*χ*^2^ × 10^−6^	*r* ^2^
VRH	17.2 ± 0.8	0.36 ± 0.08	$Bexp[{(\frac{{T}_{0}}{T})}^{0.25}]$	2.75	0.987
ES localization	0.32 ± 0.03	0.9 ± 0.1	$Bexp[{(\frac{{T}_{0}}{T})}^{0.5}]$	3.34	0.985
2DWL	307 ± 22	0.15 ± 0.04	[*G*_0_ + *G*_1_*ln*(*T*)]^−1^	2.62	0.988
3DWL	2.8 ± 0.4	0.8 ± 0.2	[*G*_0_ + *G*_1_*T*^*p*/2^]^−1^	6.11	0.928
FIT	0.080 ± 0.004	1.0 ± 0.1	${\rho }_{0}exp[\frac{{T}_{1}}{T+{T}_{0}}]$	2.13	0.991
Kondo effect	0.52 ± 0.04	0.92 ± 0.09	${\rho }_{0}ln[\frac{{T}_{0}}{T}]$	2.43	0.989

**Table 2:** Comparison between different theoretical models of *ρ* vs *T* curves for device #2.

Model	*A* (*μ*Ω*cmK*^−*n*^)	*n*	*ρ*_*I*_(*T*)-dependence	*χ*^2^ × 10^−6^	*r* ^2^
VRH	6.0 ± 0.2	0.53 ± 0.05	$Bexp[{(\frac{{T}_{0}}{T})}^{0.25}]$	7.71	0.979
ES localization	0.39 ± 0.02	0.9 ± 0.1	$Bexp[{(\frac{{T}_{0}}{T})}^{0.5}]$	7.94	0.974
2DWL	71 ± 7	0.29 ± 0.03	[*G*_0_ + *G*_1_*ln*(*T*)]^−1^	6.97	0.981
3DWL	0.7 ± 0.2	0.9 ± 0.2	[*G*_0_ + *G*_1_*T*^*p*/2^]^−1^	14.9	0.863
FIT	0.154 ± 0.004	1.0 ± 0.1	${\rho }_{0}exp[\frac{{T}_{1}}{T+{T}_{0}}]$	6.41	0.983
Kondo effect	0.50 ± 0.04	0.93 ± 0.09	${\rho }_{0}ln[\frac{{T}_{0}}{T}]$	7.04	0.980

By analyzing the values of *n*, however, it is observed that, in the case of VRH and 2DWL, they are very far from the values predicted by the theory for the different scattering mechanisms producing the metallic resistivity term. Moreover, the best fitting curves obtained with ES localization give values of *T*_0_ parameter several orders of magnitude smaller than those of aluminum compounds, which are usually characterized by a very high energy barrier.

Therefore, the only theoretical interpretations, capable to reproduce the experimental resistivity data and reasonable from a physical point of view, are: 3DWL, FIT, and Kondo effect. It is important to stress that the standard DC electrical analysis alone is not sufficient to precisely identify the exact physical origin of the transport mechanisms at work. In this respect, more sensitive measurements and an additional experimental analysis, performed with noise spectroscopy, can help in determining the cause of the observed resistivity behaviour.

### Voltage-noise spectral density measurements

In the analysis of fluctuation processes, a crucial aspect is to establish the frequency composition of the measured voltage-noise spectral density *S*_*V*_.

**Figure 2:**
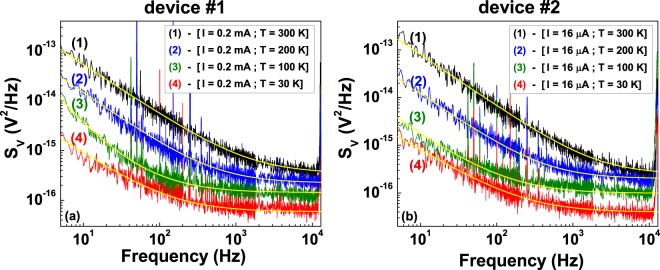
Voltage-spectral density of large-strip and small-strip devices. The frequency dependence of *S*_*V*_, at four different temperatures and at a fixed DC bias current value, is shown for device #1 (**a**) and for device #2 (**b**). The yellow solid curves are the best fit using equation (2), where the *η*_1_ and *η*_2_ parameters have been evaluated at each temperature by varying the bias current.

Figure 2 shows *S*_*V*_ at four different temperatures and at a constant bias current, for device #1 and device #2 in panels (a) and (b), respectively. Apart from a number of peaks at definite frequencies, due to external noise sources, a general trend is observed in all spectra. In particular, two distinct noise components are clearly visible: a first component, of 1/f-type, at low frequencies, and a second with constant amplitude in frequency, at higher frequencies. A general expression can be used to reproduce *S*_*V*_ as^21^

2 $${S}_{V}(f,I,T)=\frac{K(T)}{{f}^{\gamma (T)}}{I}_{DC}^{{\eta }_{1}(T)}+C(T){I}_{DC}^{{\eta }_{2}(T)},$$

where *K* is the 1/f noise amplitude, *γ* is the frequency slope, varying in the range between 0.8 and 1.2, *C* is the “white noise” amplitude, *η*_1_ and *η*_2_ the exponents of the DC bias current. All these parameters are, in principle, temperature dependent. A good agreement between equation (2) and the experimental data is shown by the yellow solid lines in Fig. 2.

More in details, the temperature dependencies of *η*_1_ and *η*_2_ allow to distinguish between the different fluctuation mechanisms.

In the case of WL effects, nonequilibrium universal conductance fluctuations are established, and a linear dependence on *I*_*DC*_ of the 1/f noise is expected together with a current-independent white noise contribution (*η*_1_ = 1 and *η*_2_ = 0)^22,23^.

The possible presence of intergranular tunneling processes modeled in terms of FIT theory is connected to a quadratic bias current dependence both for the 1/f noise and for the white noise components (*η*_1_ = *η*_2_ = 2)^24^.

When the low-temperature transport of a system is dominated by Kondo effect, standard resistance fluctuations occurs, characterized by a quadratic current dependence of the 1/f noise and a current-independent white noise, due to the Johnson thermal noise 4*k*_*B*_*TR* (*η*_1_ = 2 and *η*_2_ = 0)^21^.

**Figure 3:**
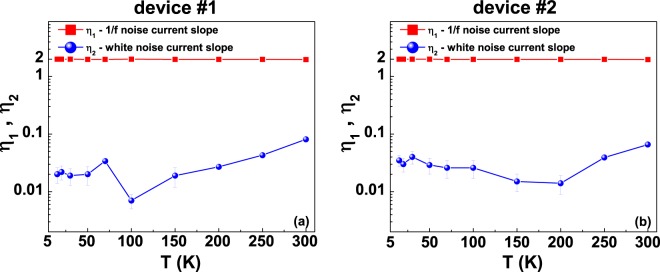
Voltage-noise current power exponents. The temperature dependencies of the bias current power exponents *η*_1_ (red squares) and *η*_2_ (blue circles) of equation (2) are shown for device #1 (**a**) and for device #2 (**b**). The lines are only guides to the eye.

The data shown in Fig. 3 are obtained from the analysis of the *S*_*V*_ temperature and current dependence, reported in Supplementary Fig. S1, and indicate that resistance fluctuations are, most probably, the cause of the measured electric noise. Therefore, the normal state transport properties of granular aluminum films could be interpreted in terms of a Kondo model, as also recently speculated and confirmed with magneto-transport investigations by Bachar *et al*.^8^.

**Figure 4:**
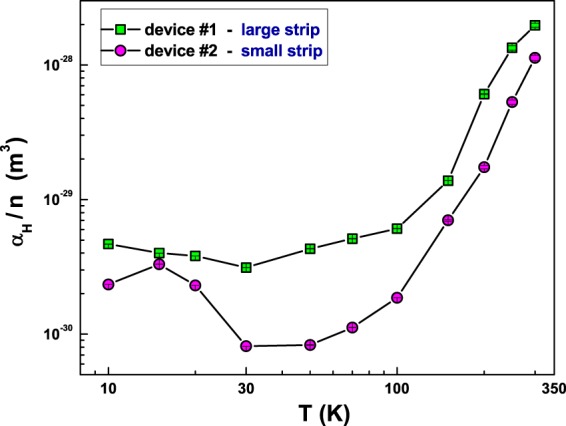
Noise level in terms of normalized Hooge parameter. Temperature dependence of *α*_*H*_/*n* parameter for the large-strip (green squares) and small-strip (violet circles) devices. An unusual noise level increase is observed below 30 K.

Once that the noise source has been attributed to resistance fluctuations, a standard approach, represented by the Hooge semi-empirical relation, can be used. The main idea behind this relation, which has no physical explanation, is that, whatever the carriers do when producing 1/f noise, they do it independently. This leads to the following expression for the 1/f noise^21^

3 $${S}_{V}=\frac{{\alpha }_{H}}{n}\frac{1}{{\rm{\Omega }}}\frac{1}{{f}^{\gamma }}{R}^{2}{I}_{DC}^{2},$$

where Ω is the sample volume, *n* is the carrier density, and *α*_*H*_ is the empirical Hooge constant; *α*_*H*_/*n* is a normalized measure for the relative noise at different temperatures, useful when the carrier density is not known. Once the noise spectral density has been measured, from equation (3) it is straightforward to compute the noise level *α*_*H*_/*n*. In Fig. 4, its temperature dependence is shown for the device #1 (green squares) and for device #2 (violet circles). As evident in the figure, the two different devices have comparable values of *α*_*H*_/*n* and show a similar temperature behaviour. In particular, a strong noise reduction occurs from 300 K down to 30 K. Moreover, below 30 K a moderate increase is observed, suggesting the occurrence of an additional fluctuation process.

**Figure 5:**
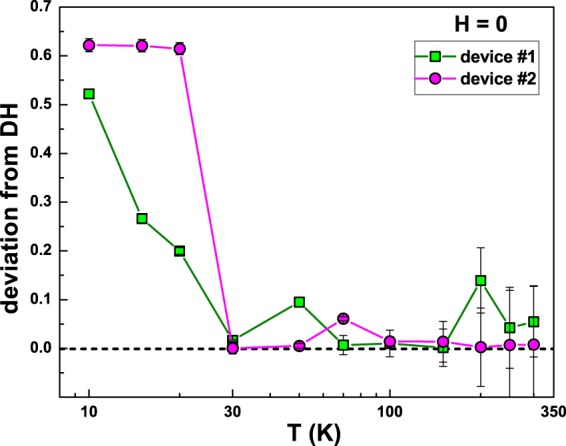
Check on the validity of the Dutta and Horn model. The deviation from DH model is experimentally estimated as the absolute value of the difference between the LHS and RHS of equation (4). The best fit to the experimental data of the RHS of equation (4) is obtained with *λ*_0_ = (1.4 ± 0.2) × 10^13^ Hz for both the devices.

One possible explanation for the observed noise temperature dependence can be identified in the, widely accepted, theory proposed by Dutta and Horn several years ago^25^. In their model, the origin of resistance fluctuations is associated to vacancy and interstitial diffusion. The number of diffusing vacancies, determined by the temperature, is responsible for the noise amplitude. At the same time, the diffusion of vacancies, which is characterized by a distribution of activation energies, accounts for the 1/f nature of the frequency spectrum. This scenario can be applied to the case of granular aluminum, where grain boundaries can act as centers of vacancy creation. The validity of this theoretical interpretation can be verified by using the Dutta and Horn (DH) relation, defined as^25^

4 $$\frac{\partial ln[\frac{{\alpha }_{H}}{n}\frac{1}{{\rm{\Omega }}}]}{\partial lnT}=1-ln(\frac{{\lambda }_{0}}{{f}^{\ast }})(1-\gamma ),$$

where *λ*_0_ is a constant of the order of phonon frequencies, and ${f}^{\ast }$ is a reference frequency usually fixed at 1 Hz. This equation relates the deviation of the noise spectrum from the linear temperature dependence to the deviation from the pure 1/f behaviour. The absolute value of the difference between the left- (LHS) and right-hand sides (RHS) of equation (4) can then be considered as a “figure of merit” for the applicability of the DH model. In Fig. 5 such “figure of merit” is shown for device #1 (green squares) and for device #2 (violet circles), indicating a significant deviation from the DH model at temperatures below 30 K. Therefore, another noise process has to be invoked at temperatures below 30 K. A possible source could be the spin-flip scattering of conducting electrons due to local magnetic moments, occurring in Kondo systems^26^.

### Magnetic feld noise dependence

As well known, the Kondo effect, suggested as the source of fluctuations by the voltage-spectral density measurements, is essentially originated by magnetic moments interacting with conduction electrons. However, according to ref.^8^, due to the high electronic density of states, the presence of magnetic impurities, for instance iron, in an aluminum matrix can be ruled out. Therefore, another possible origin for magnetic moments in granular aluminum could be found in the presence of free spins attributed to surface effects at metal/oxide interfaces or to a volume effect^27,28^. This interpretation has already been used to explain a negative magnetoresistance (MR) in nanoscale granular aluminum films, observed at low temperatures (below 30 K) with high-intensity magnetic fields (higher than 5 T)^8^.

So far noise measurements in high magnetic field (*H*) have never been done, due to the strong additional noise induced by the field in the sample and connecting wires through mechanical vibrations. However, noise spectroscopy is a technique more sensitive than conventional DC electric transport. Therefore, a possible negative magneto-noise effect could be expected also with weak applied magnetic fields, which are compatible with the noise measurement technique.

**Figure 6:**
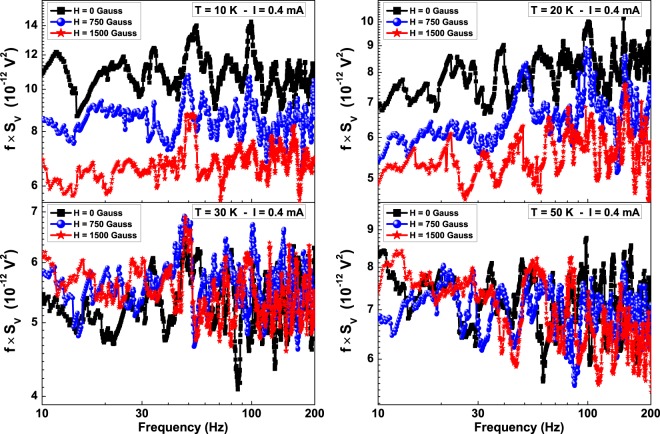
Magnetic field dependence of the normalized 1/f noise component for device #2. The low-frequency normalized 1/f noise is shown at four different temperatures, between 10 and 50 K, by fixing the bias current at 0.4 mA. The applied external magnetic field, up to 1500 Gauss, has an orientation parallel to the sample surface and perpendicular to the current in the sample.

The results of such measurements are shown in Fig. 6, where the normalized 1/f noise *fS*_*V*_, proportional to *K* for fixed bias current, is reported for the device #2 at *T* = 10, 20, 30 and 50 K and for *H* = 0, 750 and 1500 Gauss. A noise level reduction with *H* is observed at 10 K and at 20 K (see upper panels in Fig. 6), while no effect associated with *H* is found at 30 K and at higher temperatures (see lower panels in Fig. 6). This suggests that in the low-temperature region, where the Kondo effect is visible, the magnetic field seems to have an ordering effect that affects the charge carrier fluctuations.

**Figure 7:**
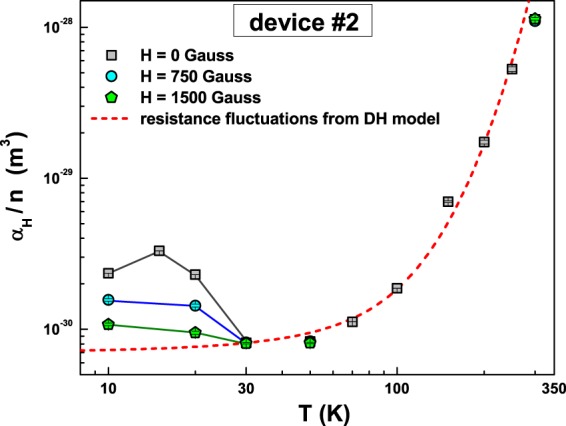
Relative noise level as a function of the external magnetic field. The temperature dependence of the normalized Hooge parameter *α*_*H*_/*n* is shown at three different external magnetic fields. The noise amplitude values for device #2, obtained with the Dutta and Horn resistance fluctuations model, are reported as dashed red line.

This result can also be seen by looking at the temperature dependence of the normalized Hooge parameter *α*_*H*_/*n* by varying *H*. In Fig. 7, this is shown together with the value predicted by the DH model equation (4). From the figure, it is clear that the DH model (red dashed line) well reproduces the experimental data above 30 K and represents the lower limit of the 1/f noise below 30 K. The additive low-temperature fluctuations can be reduced by an external magnetic field (see blue circles and green pentagons in Fig. 7) and completely suppressed for values of $H\ge 2000$ Gauss. This suggests that the observed excess noise below 30 K is of magnetic origin and is related to interface magnetic moments, probably formed at the Al/Al oxide interface. This scenario is compatible with the logarithmic resistance increase at low temperatures and with a negative MR effect, previously measured on similar samples^8^.

In order to investigate the possibility of a magnetic phase transition occurring in the vicinity of 30 K, SQUID measurements on high-resistive AlO_*x*_ samples are currently in progress. However, it is here interesting to underline that an analogous phenomenon of noise reduction induced by polarized DC current has been found in disordered manganite thin films. In that case, a dominant role is played by the spin torque of interface magnetic moments, formed in the grain boundary regions^29^.

### Discussion and possible implications for the superconductive state

Once that the low-temperature magnetic nature of the fluctuations has been identified, it would be useful to make an estimation of the number of fluctuators. As already reported for disordered metallic thin films, which are characterized by an excess 1/f noise due to oxygen impurities and strongly related to grain size effects, the temperature-dependent number of “noisy” defects can be evaluated as: $N(T)={R}_{\ast }^{2}(T){\beta }^{-2}$^30^. Here, the scattering power *β* for various point defects and impurities in most metallic compounds is fixed to 1 *μ*Ω, while the resistance noise magnitude ${R}_{\ast }^{2}$ is expressed as^30^

5 $${R}_{\ast }^{2}(T)=\frac{{\alpha }_{H}}{n}\frac{1}{{\rm{\Omega }}}{\{\frac{{R}_{300}}{R(T)}[R(T)-{R}_{300}]\}}^{2},$$

where *R*_300_ is the room-temperature resistance value. Using equation (5) for device #2, the number of fluctuators is *N* ≈ 1.3 × 10^4^ at 10 K (the lowest investigated temperature for noise analysis) and *N* ≈ 1.8 × 10^2^ at 30 K. These values are much smaller than the number of grains in the investigated volume, that can be estimated as *N* ≈ 1 × 10^8^, by assuming spherical grains with an average size of 4 nm + 1 nm oxide. This suggests, therefore, that only a fraction of the overall defects giving rise to a measurable resistivity upturn is also unstable and generates a measurable 1/f noise. Nonetheless, these unstable defects represent a very sensitive probe for the occurrence of the Kondo effect and of its magnetic dependence.

From equation (5) it is clear that the number of fluctuators is proportional to the normalized noise level *α*_*H*_/*n*. This would imply that, by looking at Fig. 7, at low temperatures and in correspondence to the occurrence of the Kondo effect, there is an increase of fluctuators. However, it is not possible to exclude another explanation, that the occurrence of the magnetic inherent scattering associated to the Kondo effect enhances the amplitude of the resistance fluctuations while not changing the number of active fluctuators. In all cases described, the common experimental evidence is a noise reduction due to an external magnetic field, see Fig. 7.

**Figure 8:**
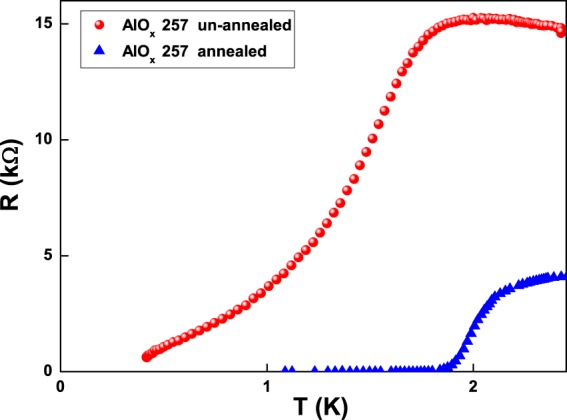
Annealing effect near the superconducting transition. *R* vs *T* curves at temperatures below 2.5 K are shown for a typical investigated sample before (red circles) and after (blue triangles) the annealing process. During heat treatment, the normal state resistance dropped from 5.5 kΩ to about 4.3 kΩ at room temperature, thus changing from a non-superconducting (above 300 mK) to a superconducting state below 1.8 K. These measurements have been performed by using a ^3^He cryostat with an operational range down to 300 mK. It is interesting to note that the sheet resistance of the annealed film does practically not change from 300 K down to 2.5 K, while the un-annealed sample shows an increase to ~15 kΩ.

The recent discovery of ferromagnetism in nonmagnetic oxides, such as CeO_2_, Al_2_O_3_, ZnO, In_2_O_3_, and SnO_2_^31,32^, strongly supports the magnetic origin of the low-temperature charge carrier fluctuation mechanism. In particular, it is assumed that the exchange interactions between interface localized electron spin moments, resulting from the oxygen vacancies, are the origin of ferromagnetism in these materials. The nature of these exchange interactions is still not clear, but one may expect that electrons trapped in oxygen vacancies are polarized to give a ferromagnetic effect^31^. Therefore, it is possible to speculate that the reduction of oxygen vacancies in granular films could reduce the spin density and, consequently, the scattering mechanism of magnetic moments with conduction electrons, responsible for the Kondo effect. A way to decrease the oxygen content is an annealing process. As shown in Fig. 8, un-annealed granular aluminum films can have high resistance values and are characterized by a resistance uprise before the superconducting transition (see red circles in Fig. 8). By annealing the samples, for 15 min at 250 °C and another 2 min at 300 °C in air^6^, a resistance decrease with the disappearance of the Kondo-like conductivity (see blue triangles in Fig. 8) it is observed. When a pure metallic conduction is restored, data reported in literature for various disordered metal films show that no noise increase occurs at low temperatures and no magnetic field dependence is found for the 1/f component^21,25^.

The enhancement of the superconducting critical temperature in annealed samples, visible in Fig. 8, suggests a possible relation between superconductivity and the presence of a spin-flip scattering of conducting electrons. How this coexistence is possible is an intriguing issue and remains still unresolved. Noise spectroscopy may be useful to shed light on this phenomenology, providing information on a possible unconventional mechanism for superconductivity in granular films. Therefore, further theoretical and experimental work is necessary.

## Conclusions

Transport and noise measurements in granular aluminum thin films support the idea that at high temperature the carrier transport is dominated by charge impurity resistivity contributions. At low temperatures, an upturn of resistivity is observed, which has been attributed to several possible mechanisms. The noise spectroscopy results strongly indicate that the Kondo effect, conventionally ascribed to magnetic impurities, is responsible of the observed behaviour. The magnetic field dependence of noise amplitude suggests that the interface magnetic moments, probably formed at the Al/Al oxide interface, are the main fluctuators. This could be of importance also at lower temperatures and in the superconducting state, as a possible source of decoherence in systems employing this material.

## Methods

**Figure 9:**
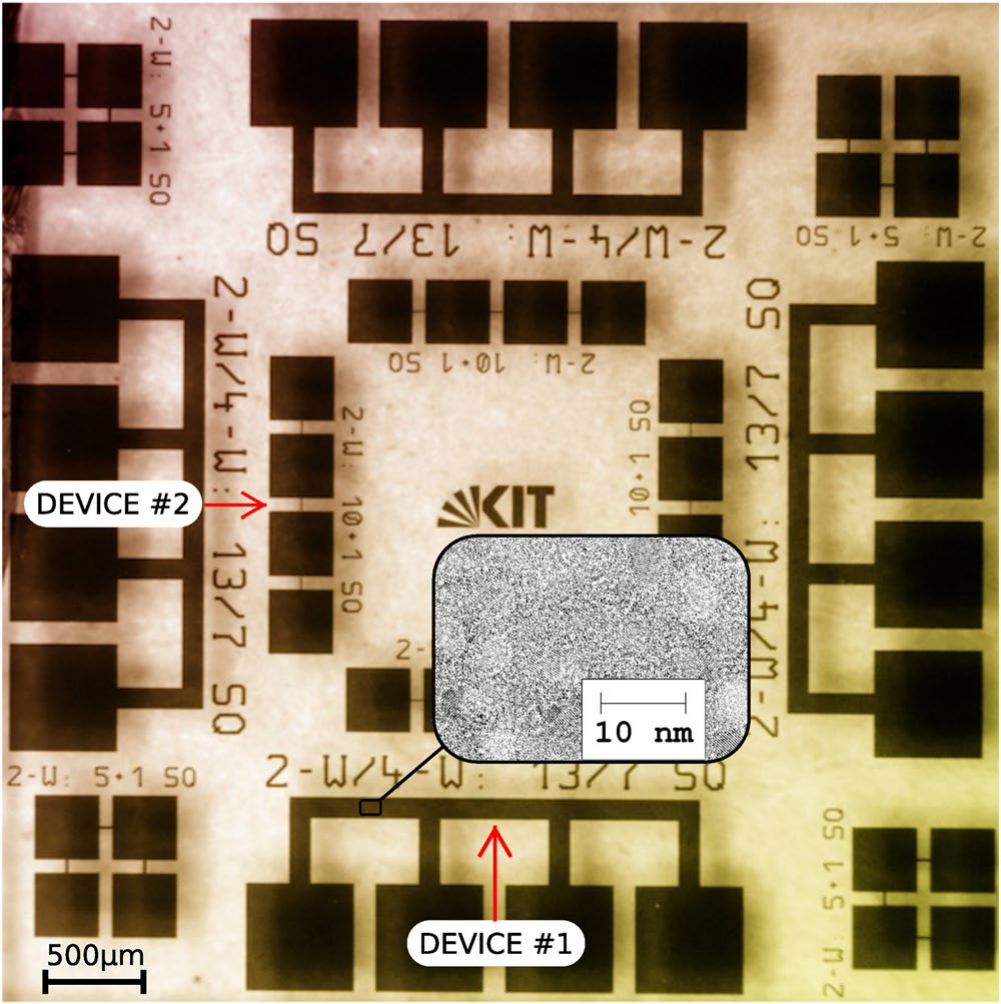
Optical image of the fabricated granular aluminium oxide sample. The inset shows a TEM micrograph with the typical granular structure of films with a similar sheet resistance^6^. The average grain size is about 4 nm. The four-probe contact configuration is shown for the two investigated devices (large-strip device #1 and small-strip device #2).

Granular aluminum oxide thin film samples of a thickness of 20 nm were grown at room temperature by DC magnetron sputter deposition onto a sapphire substrate. The details of the fabrication process are reported in ref.^6^, where also the reproducibility and stability of the sheet resistance of different samples as well as their kinetic inductance in the superconducting state are discussed. However, here slightly different process conditions were used. In order to remove contaminants from the substrate surface prior to the AlO_*x*_ sputter deposition, the samples were cleaned using a pulsed DC Ar plasma for 1 min at a power of 20 W. The pulsed DC AlO_*x*_ sputter process operated at a power of 150 W, a repetition rate of 50 kHz, and an Ar/O_2_ pressure of 3 *μ*bar. The sample was patterned using an optical resist mask, producing 5 × 5 mm^2^ chips, with a layout shown in Fig. 9. The experimental results of two different devices are here reported. Device #1 is a strip of length 500 *μ*m and width 100 *μ*m. The voltage was measured through two striplines of length 300 *μ*m and width 100 *μ*m. These contributed to overall noise even if there was no current flowing. Device #2 is a strip of length 60 *μ*m and width 6 *μ*m. The voltage was measured through two square pads of size 300 *μ*m × 300 *μ*m. These had a negligible contribution to overall noise.

Electric transport measurements were performed in a closed-cycle refrigerator with temperature stabilization between 300 and 8 K. Low-noise DC and AC electronic bias and readout were used. In particular, the AC signal was amplified with a Signal Recovery 5113 preamplifier, analyzed with a dynamic signal analyzer type HP35670A, and acquired with an automated LabVIEW software. The possible presence of spurious noise sources, essentially due to wire bonding contacts, was ruled out by resorting to a dedicated experimental procedure, described in ref.^33^. A dipole electromagnet, type 3470 from GMW Associates, was used for the generation of the external magnetic field. The gap between the coils, fixed by the experimental setup constraint, allows to reach a maximum magnetic field value of 1500 Gauss at the maximum operating current of 3.5 A without water cooling.

## Electronic supplementary material

Supplementary Information
