# Author Correction: Kondo-like transport and magnetic field effect of charge carrier fluctuations in granular aluminum oxide thin films

**DOI:** 10.1038/s41598-018-33003-y

**Published:** 2018-10-23

**Authors:** C. Barone, H. Rotzinger, C. Mauro, D. Dorer, J. Münzberg, A. V. Ustinov, S. Pagano

**Affiliations:** 1 Dipartimento di Fisica “E.R. Caianiello” and CNR-SPIN Salerno, Università di Salerno, I-84084 Fisciano Salerno, Italy; 2 Physikalisches Institut, Karlsruhe Institute of Technology, 76131 Karlsruhe, Germany; 3 Dipartimento di Ingegneria, Università del Sannio, I-82100 Benevento, Italy; 4 Russian Quantum Center, National University of Science and Technology MISIS, 119049 Moscow, Russia

Correction to: *Scientific Reports* 10.1038/s41598-018-32298-1, published online 17 September 2018

The Acknowledgements section in this Article in incomplete.

“S. Abate of CNR-SPIN Salerno is acknowledged for his valuable technical support. This work is partially supported by University of Salerno through grants FARB15PAGAN and FARB16CAVAL.”

should read:

“S. Abate of CNR-SPIN Salerno is acknowledged for his valuable technical support. This work is partially supported by University of Salerno through grants FARB15PAGAN and FARB16CAVAL.

A.V.U. acknowledges partial support from the Ministry of Education and Science of the Russian Federation in the framework of Increase Competitiveness Program of the National University of Science and Technology MISIS (Contract No. K2-2017-081).”

