# Supplementary material for: Kondo-like transport and magnetic field effect of charge carrier fluctuations in granular aluminum oxide thin films
Source: Sci Rep. 2018 Sep 17;8:13892. doi: 10.1038/s41598-018-32298-1 (PMC6141613; doi:10.1038/s41598-018-32298-1)
Supplement: Supplementary file 1 — Supplementary Information [file 41598_2018_32298_MOESM1_ESM.pdf]

Supplementary Information for "Kondo-like transport and magnetic field effect of charge carrier fluctuations in granular aluminum oxide thin films"

C. Barone[1],* H. Rotzinger[2], C. Mauro[1,3], D. Dorer[2],

J. Münzberg[2], A. V. Ustinov[2,4], and S. Pagano[1]

[1]*Dipartimento di Fisica "E.R. Caianiello" and CNR-SPIN Salerno, Università di Salerno, I-84084 Fisciano, Salerno, Italy*

[2]*Physikalisches Institut, Karlsruhe Institute of Technology, 76131 Karlsruhe, Germany*

[3]*Dipartimento di Ingegneria, Università del Sannio, I-82100 Benevento, Italy*

[4]*Russian Quantum Center, National University of Science and Technology MISIS, 119049 Moscow, Russia*

*Electronic address: cbarone@unisa.it

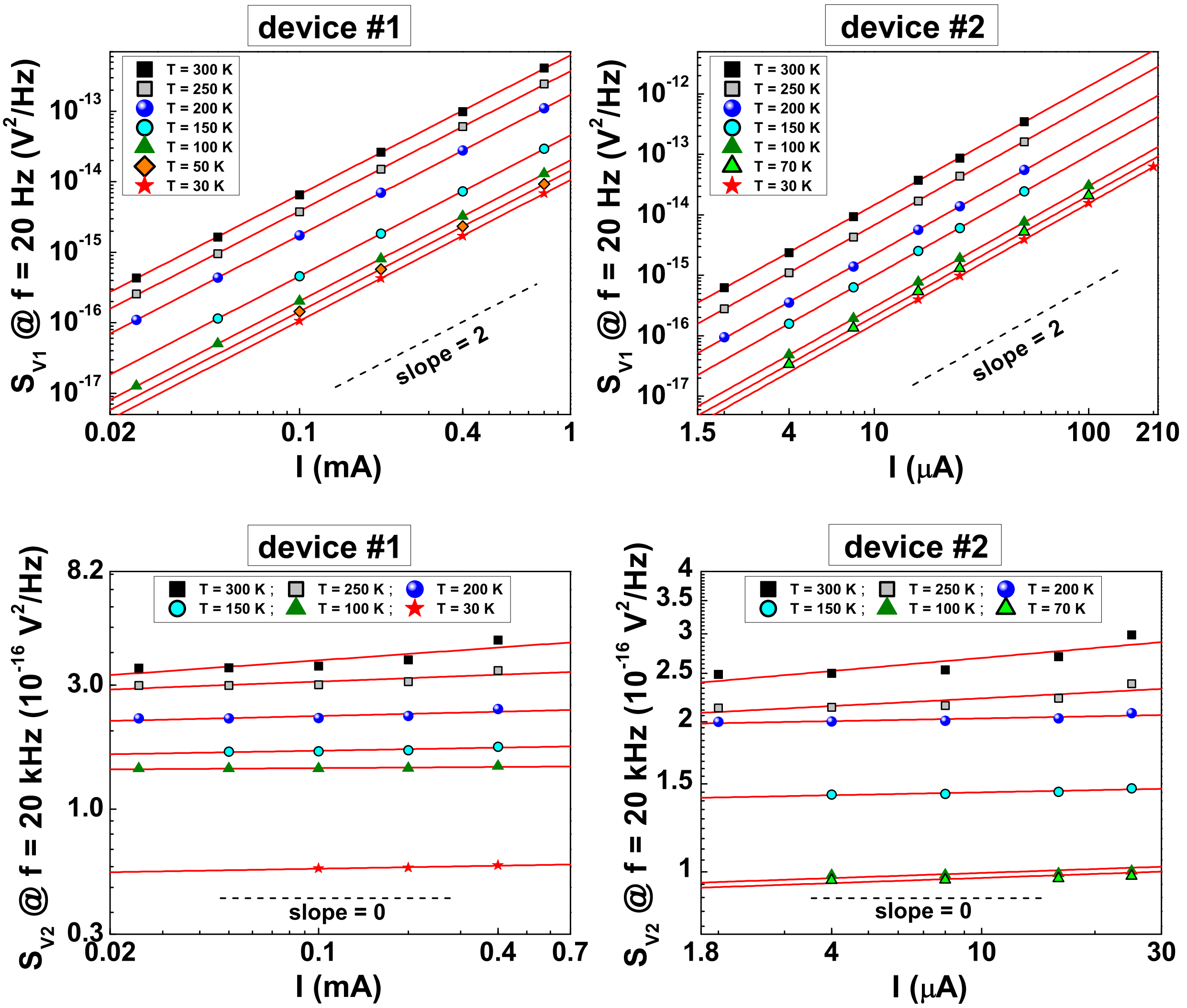

Fig. S1: **Analysis of the current dependence of** S_V**.** The current dependencies of 1/f (upper panels) and white-noise (lower panels) components of the measured voltage-noise spectral density are shown for device #1 (left panels) and for device #2 (right panels).
